# Using a ligate intestinal loop mouse model to investigate *Clostridioides difficile* adherence to the intestinal mucosa in aged mice

**DOI:** 10.1371/journal.pone.0261081

**Published:** 2021-12-22

**Authors:** Pablo Castro-Córdova, María José Mendoza-León, Daniel Paredes-Sabja

**Affiliations:** 1 Facultad de Ciencias de la Vida, Microbiota-Host Interactions and Clostridia Research Group, Departamento de Ciencias Biológicas, Universidad Andrés Bello, Santiago, Chile; 2 ANID-Millennium Science Initiative Program—Millennium Nucleus in the Biology of the Intestinal Microbiota, Santiago, Chile; 3 Department of Biology, Texas A&M University, College Station, Texas, United States of America; Cornell University, UNITED STATES

## Abstract

Interaction of *Clostridioides difficile* spores with the intestinal mucosa contributes to the persistence and recurrence of the infection. Advanced age is one of the main risk factors for *C*. *difficile* infection and recurrence of the disease. However, interaction of *C*. *difficile* spores with the intestinal mucosa during aging has not been evaluated. In the present work, using intestinal ligated loop technique in a mouse model, we analyzed *C*. *difficile* spore adherence and internalization to the ileum and colonic mucosa during aging. Additionally, we provide visual documentation of the critical steps of the procedure. Consequently, our data suggest that spore internalization in the ileum and colonic mucosa is higher in elderly mice rather than adults or young mice. Also, our data suggest that spore adherence to the ileum and colonic mucosa decreases with aging.

## Introduction

*Clostridioides difficile* is a Gram-positive, anaerobic, and spore former bacterium and the leading pathogen causing hospital acquiring diarrhea associated with antibiotics [1,2]. The *C*. *difficile* infection (CDI) is characterized by the manifestation of diarrhea that can produce mild to watery diarrhea, abdominal pain, and tenderness [3]. In severe cases, patients can become dehydrated or produce toxic megacolon [3]. CDI is lethal in ~5% of infected patients [4,5]. The ~15–30% of CDI-recovered patients manifest recurrent CDI (R-CDI) [5,6].

The two main risk factors for CDI are the continuous alteration of the intestinal microbiota caused by antibiotics, and the age over 65 years old [7,8], being the 91% of the CDI deaths in this age group [9]. This increasing association of CDI with aging could be explained by age-related physiologic changes such as the immunosenescence and age-related dysbiosis of the intestinal microbiota; thus, reducing protection against *C*. *difficile*.

The immunosenescence is characterized by a progressive decrease in the immune system’s effectiveness associated with aging, increasing susceptibility to infections in older adults due to the impaired innate and adaptive immune response [10]. Including dysfunctional antigen-presenting cells, reduced chemotaxis to inflammatory stimuli of natural killer cells, neutrophils [11,12], reduced activity in bacterial phagocytosis by monocytes and macrophages [12,13]. There is also a reduced antibody response to exogenous antigens and vaccines by B-cells [14]. These changes may be explained by altered intracellular communication, telomere attrition, epigenetic alterations in the earliest hematopoietic stem cells [14]. Therefore, elderly patients have an increased risk of bacterial infections such as CDI.

Age-related dysbiosis is characterized by changes in species diversity, becoming enriched in pro-inflammatory commensal species [15]. In particular, a decline of *Bifidobacterium* [16] and Clostridiales with enrichment in Proteobacteria and an overrepresentation of Enterobacteriaceae [14]. Age-related dysbiosis is associated with a reduced protective role of the microbiota against CDI [17,18]. For example, it has been reported that fecal emulsions from geriatrics patients have low inhibitory activity in the growth of *C*. *difficile in vitro* compared with fecal emulsions from healthy adults [17].

During CDI, *C*. *difficile* forms metabolically dormant spores that are essential for R-CDI [19]. Accordingly, with this observation, recently, by using the surgical procedure of intestinal ligated loop, we demonstrate that *C*. *difficile* spores adhere [20] and internalize into the intestinal mucosa contributing to R-CDI [21]. However, whether aging affects the adherence and internalization of *C*. *difficile* spores to the intestinal mucosa remains unclear. Due to the varieties of critical points during the ligated loop procedure (i.e., tissue manipulation and sample mounting), in the present work, we provide attached visual documentation describing the critical steps to acquire high-resolution confocal images and quantify spore adherence and internalization in the intestinal mucosa. Using this technique, we evaluated spore adherence and internalization into the intestinal mucosa of young (7-weeks-old), adult (1-year-old), and elderly mice (2-years-old). Our results suggest that spore adherence decreases in aged ileum and colonic mucosa, whereas spore entry into intestinal epithelial cells of the intestinal mucosa is increased. These results suggest that the increased spore entry may contribute to the high recurrence rates of CDI in elderly patients.

## Materials and methods

The step-by-step protocol is in the following link https://dx.doi.org/10.17504/protocols.io.bvzin74e

### Mice

C57BL/6 (male or female) 7-weeks (*n* = 5), 1-year-old (*n* = 4) and 2-years-old (*n* = 4) were obtained from the breeding colony at Departamento de Ciencias Biológicas, Universidad Andrés Bello derived from Jackson Laboratories. Mice were housed with *ad libitum* access to food RMH 3000 (Prolab, USA) and autoclaved distilled water. Bedding and cages were autoclaved before use. Mice were housed with 12-h cycle of light and darkness at 20–24°C with 40–60% humidity. All mice employed in this study were previously assayed for the absence of *C*. *difficile* in the feces.

### Ethics statement

All procedures complied with ethical regulations for animal testing and research. This study received ethical approval from the Animal Care and Use Committee of the Departamento de Ciencias Biológicas of the Universidad Andrés Bello (Protocol 0038/2018). Details of animal welfare and steps taken to ameliorate suffering are included in the methods section of the manuscript.

### Purification of *C*. *difficile* spores and inoculum preparation

*C*. *difficile* spores strain R20291 (CM210) were purified as described previously [20]. Briefly, a 1:1,000 dilution of an overnight culture of *C*. *difficile* in BHIS medium (3.7% brain-heart infusion broth (BD, USA) supplemented with 0.5% yeast extract (BD, USA) and 0.1% L-cysteine (Merck, USA)) was plated onto agar plates with 70:30 sporulation medium that was prepared as follow: 6.3% bacto peptone (BD, USA), 0.35% proteose peptone (BD, USA), 0.07% ammonium sulfate (NH_4_)_2_SO_4_ (Merck, USA), 0.106% Tris base (Omnipur, Germany), 1.11% brain heart infusion extract (BD, USA), and 0.15% yeast extract (BD, USA), 1.5% Bacto agar (BD, USA). Plates were incubated for 7 days at 37°C. All this procedure was performed in a Bactron300 anaerobic chamber (Shellab, OR, USA). Then the colonies were scraped out with ice-cold-sterile distilled milli-Q water and were washed 5 times by resuspension and centrifugation at 18,400 × *g* for 5 min each. Spores were separated by gradient density using 45% Nicodenz and centrifugated at 18,400 × *g* for 40 min. The spore pellet was washed 5 times and resuspended in ice-cold sterile milli-Q water and centrifugation at 18,400 × *g* for 5 min to remove Nycodenz. Spores were counted in Neubauer chamber, adjusted at 5 × 10^9^ spores/mL, and stored at –80°C until use. To prepare the spore inoculum, 100 μL of the spore stock was centrifugated at 18,400 × *g* for 5 min, resuspended in 100 μL of saline solution, and loaded in low-dead volume tubercule syringes under a biosafety cabinet.

### Surgery

The surgical procedure was performed under aseptic conditions. Surgical material was sterilized by autoclave, and all the surfaces were sanitized with 1:10 dilution of commercial household bleach and 70% ethanol. Personal protective equipment was used during the surgery, such as a disposable laboratory coat, goggles, gloves, cap, and mask.

Male or female, 18–25 g C57BL/6 mice of 8–12 weeks were fasted overnight (15 h) before the surgery with free access to water. On the next day, depth anesthesia was induced with 4% (vol/vol) isoflurane with a flow of 0.6 L/min using an isoflurane induction chamber (RDW, USA). Then the mouse was put prone in a stainless-steel surgical tray, and 2% (vol/vol) isoflurane was administered using an isoflurane mask. To avoid hypothermia, a heating pad was placed under the surgery bed. To avoid corneal drying, ophthalmic drops were used. In the supine position, depth anesthesia was evaluated by hind limb toe pinch. Then the abdominal area was cleaned with 70% ethanol, shaved, and cleaned with povidone-iodine (S1A Fig, S1 Video). A midline laparotomy of ~2cm was performed, incising in the linea alba (S1B and S1C Fig, S2 Video). Using forceps Dumont N° 5 and surgical suture, sections of ~1.5 cm of the ileum and proximal colon were ligated, where blood vessels are finely separated from the ileum wall (S2A–S2C Fig), having care of not puncture or ligate the blood vessels.

To avoid inoculum loss or spore splashing during the spore injection in the loops, once the needle of a tuberculin syringe with 5 × 10^8^ *C*. *difficile* spores in 100 μL of saline was injected into the ileal through the untied side, and the knot was closed with the needle inside. Next, the inoculum containing spores was released inside the intestinal loop and the syringe removed, and the ligation was closed with a simple double knot (S3 Video). Upon loop closure, the intestines were returned to the abdominal cavities, and the incision was closed by continuous or interrupted suture (S4 Video). Then mice were removed from the isoflurane and recovered near a heat lamp with free access to water (S4 Video). Animals were monitored every 30 min for 5 h.

### Necropsy and tissue collecting

After 5 h incubation, depth anesthesia was induced by 4% isoflurane inhalation, as indicated above, and confirmed by non-response to hind limb toe pinch, then cervical dislocation was performed. The abdominal cavity was opened, and the ligated ileum and colonic loops were removed by cutting at ~5 mm from the outside of the ligatures. (S5 Video).

### Tissue fixation

In the biosafety cabinet, the ligatures were removed, and the intestines were longitudinally opened with scissors, and washed by immersion in PBS drops over a petri dish three times. Next, the tissues were fixed flat over a filter paper, imbibed with 30% sucrose in PBS–4% paraformaldehyde with the muscular layer downwards and the luminal side upwards. Stretch the tissues on the filter paper with the fixing solution and add fixing solution directly over the tissues 3 times each 5 min. Tissues were then placed in microcentrifuge tubes with 30% sucrose in PBS–4% paraformaldehyde and incubated overnight at 4°C (S6 Video). This facilitates tissue mounting and microscopy visualization.

### Immunofluorescence

Tissues were washed twice with PBS for ~5 min each at RT. Then a section of ~5 mm × 5 mm was cut. Tissues were permeabilized with PBS–0.2% Triton X-100 and incubated for 2 h at RT. Then tissues were washed three times with PBS for ~3 min each in an orbital shaker with 60 RPM at RT and incubated with blocking solution: PBS–3% BSA for 3 h at RT with an orbital shaker at 60 RPM. To immunostaining *C*. *difficile* spores and actin cytoskeleton, tissues were incubated with 1:1,000 chicken primary antibody anti-*C*. *difficile* spore IgY batch 7246 (AvesLab, USA) and 1:150 phalloidin Alexa-Fluor 568 (A12380 Invitrogen, USA); in PBS–3% BSA overnight at 4° C. This antibody does not immunoreact with epitopes of vegetative cells or with murine microbiota [21,22]. On the next day, tissues were washed and incubated with 1:350 secondary antibodies goat anti-chicken IgY Alexa Fluor-647 (ab150175, Abcam, USA) and 4.5 μg/mL of Hoechst 33342 (ThermoFisher, USA) and incubated for 3 h at RT in an orbital shaker with 60 RPM at RT. Then the tissues were washed with PBS and mounted.

### Sample mounting

Samples were mounted with the luminal side up. To identify the luminal and muscular side of tissues, samples were placed in a clean glass slide and visualized in a light-upright microscope with 20× or 40× magnification coupled to epifluorescence with a blue filter to visualize Hoechst 33342 staining. When the ileum and colon are in the correct orientation, villi or colonic crypts are visualized (S3A and S3B Fig). Tissues were placed over a glass slide with 5 μL of fluorescent mounting medium (Dako) and then 15 μL of fluorescent mounting medium over the tissues. Place a coverslip over the samples and seal with Scotch transparent tape to avoid sample drying (S7 Video). Then samples were stored in a wet chamber at 4°C until confocal visualization.

### Confocal microscopy

A confocal microscope Leica SP8 (Leica, Germany) of the Confocal Microscopy Core Facility, Universidad Andrés Bello was used to acquire images. To evaluate spore adherence and internalization in the mice intestinal mucosa, images were acquired using the objective HPL APO CS2 40× oil, numerical aperture 1.30. For signals detection, three photomultipliers (PMT) spectral detectors were used; PMT1 (410–483) DAPI, PMT2 (505–550), Alexa-Fluor 488, and PMT3 (587–726) Alexa-Fluor 555. Emitted fluorescence was split with dichroic mirrors DD488/552. Images of 1,024 × 1,024 pixels were acquired with 0.7-μm *z*-step size. Representative images were represented by three-dimensional (3D) reconstructions of intestinal epithelium using the plug-in 3D Projection of ImageJ software (NIH, USA). Villi and crypts were visualized by Hoechst and phalloidin signals.

## Results

### Quantification of spore adherence and internalization in the intestinal mucosa

**Fig 1:**
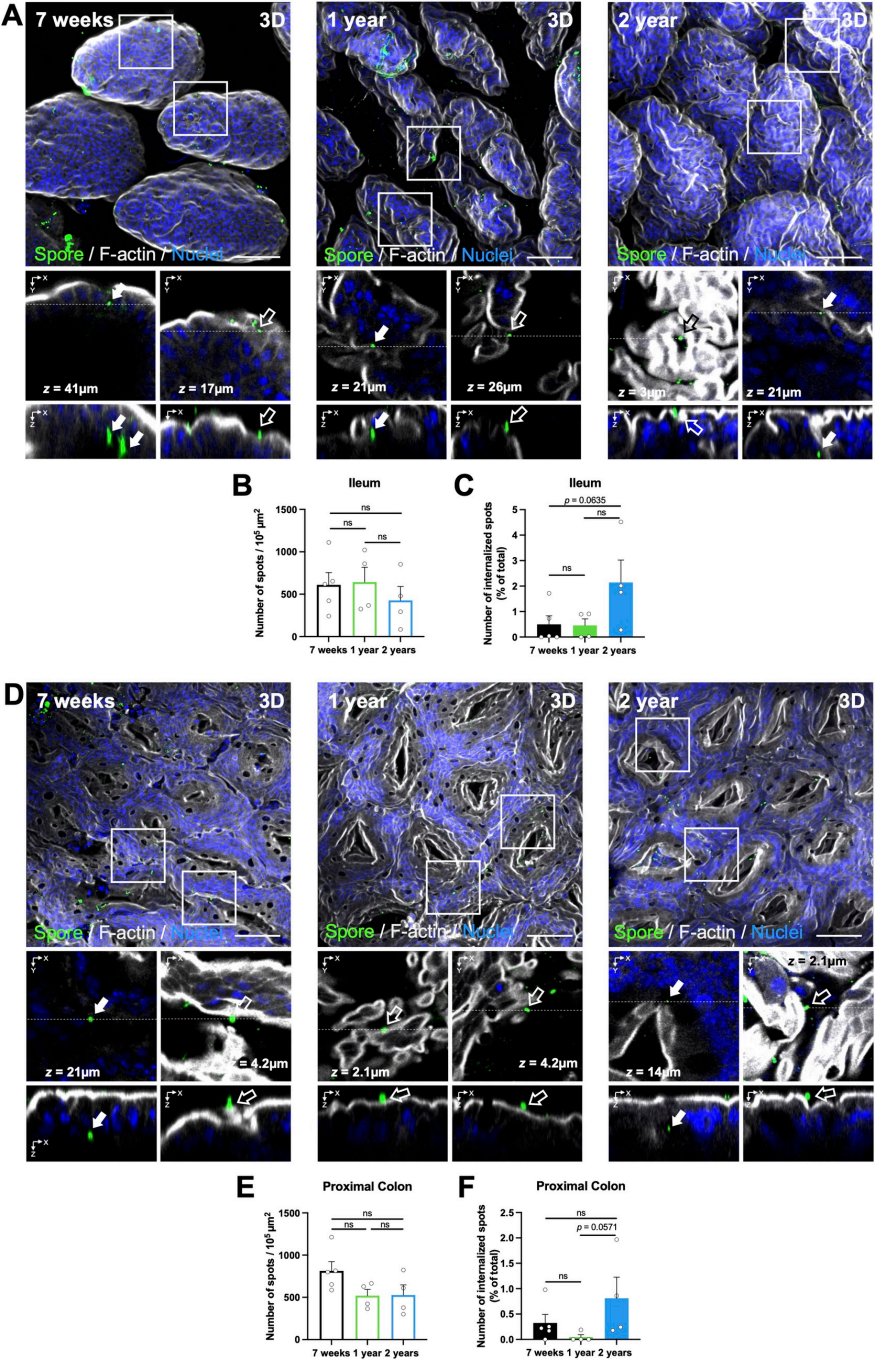
Visualization and quantification of adhered and internalized *C*. *difficile* spores in the ileum and colonic mucosa during aging. Representative confocal micrographs of (A) ileum and (D) colonic mucosa of the ligated loop. *C*. *difficile* spores are shown in green, F-actin in grey, and nuclei in blue (fluorophores colors were digitally reassigned for a better representation). The white arrow and empty arrow denote internalized and adhered *C*. *difficile* spores, respectively. Quantification of (B) adhered spots (spores) per 10^5^ μm^2^ and (C) percentage of internalized spots in the ileum or (E, F) colonic mucosa. Error bars indicate the average ± SEM. Scale bar, 50 μm. Statistical analysis was performed by two-tailed Mann–Whitney test; post-Dunn’s; test; ns, *p* > 0.05.

Confocal images were analyzed using ImageJ. First, we analyzed the spore adherence in the ileum mucosa in mice of 7-weeks-old, 1-, and 2-years-old. Representative confocal images are shown in Fig 1A. Adhered spores were considered fluorescent spots in narrow contact with actin cytoskeleton (visualized with F-actin). Adhered *C*. *difficile* spores were counted one-by-one using the plug-in Cell Counter or Point Tool of ImageJ. We observed that spore adherence varies between animals of each group and decreases according to aging. The average spore adherence was ~610, ~571, and ~427 spores, every 10^5^ μm^2^ in the ileum of mice with 7-weeks, 1-, and 2-years-old, respectively, with no significant differences between the groups (Fig 1B). We identified internalized spores using the plug-in Orthogonal View of ImageJ. Internalized spores were considered as fluorescent spots inside the actin cytoskeleton in the three spatial planes (XY, XZ, YZ) [21,23] (Fig 1A see magnifications XY and XZ). We observed that spore internalization was about ~0.5%, ~0.3%, and ~2.1% of the total spores in mice of 7 weeks old, 1- or 2 -year-old respectively, with a tendency to increase spore entry in mice of 2-years-old compared to mice of 7-weeks-old (Fig 1C).

Using the same strategy, we analyzed spore adherence to the colonic mucosa. Representative images are shown in Fig 1D. We observed a decrease in spore adherence according to the aging increase. The spore adherence was on average of ~713, 520, and 527 spores, every 10^5^ μm^2^ in the tissue of mice with 7-weeks-old, 1-year-old, and 2-years-old, respectively (Fig 1E). Results demonstrate that about 0.36, 0.05, and 0.81% of the total spores were internalized in the colonic mucosa, and we observed an increase in the spore internalization of mice of 2-years-old compared to mice of 1-year-old (*p* = 0.0571; Fig 1D magnifications XY and XZ and Fig 1F). Altogether, these data suggest that *C*. *difficile* spore adherence decreases with increased aging; by contrast, spore internalization increased in 2-years-old mice in both ileum and colonic mucosa.

## Discussion

The intestinal ligated loop technique was first described in 1953 in rabbits [24], and since then has been widely used [25–28] in several animal species such as, mouse, [29], rat [30], chicken [31] and pig [32] to study interactions and pathogenesis of bacterial pathogens with the host. Studies include those with the pathogens such as *Clostridium perfringens* [33], *Vibrio cholerae* [34], *Listeria monocytogenes* [35], and *C*. *difficile* toxins TcdA and TcdB [25–28]. However, a step-by-step protocol providing all the necessary technicalities involved in intestinal loops in mice to obtain reproducible results with reduced mortality during the procedure is missing.

In this work, we provide a detailed method with video, to quantify spore adherence and internalization in the ileum and colonic mucosa of mice, in particular, we detailed a surgical procedure of intestinal ligated loop technique, including animal anesthetize, opening of peritoneal cavities, the performance of ligated intestinal loop with inoculation of *C*. *difficile* spores, incision suturing and animal necropsy, followed by fixation of the tissues to whole-mounted tissue immunofluorescence and mounting of the sample for visualization by confocal microscopy.

Using the intestinal ligated loop technique, here, we describe that adherence of *C*. *difficile* spores to the ileum, and colonic mucosa is decreased in mice of 1-years-old and 2-years-old compared to 7-weeks-old mice. Also, that *C*. *difficile* spore entry is increased in 2-year-old mice. This finding, coupled with our recent observations that *C*. *difficile* spore entry is associated with R-CDI rates [21], suggests that increased R-CDI rates observed in elderly patients [5,6], might be linked to increased *C*. *difficile* persistence. During aging, several physiological changes occur in the intestinal mucosa that could affect spore adherence and internalization. In mice, it has been reported that a reduction of about 6-fold in the thickness of the colonic mucus layer in older mice compared to young mice [36], which enables a direct contact of bacteria with the intestinal epithelium and increased bacteria penetration [36,37]. Additionally, studies using human biopsies of older adults have reported an increased intestinal permeability due to a reduced transepithelial electric resistance compared to young humans [38], being those changes involved in permeability to ions but not to macromolecules [39]. Recently we have demonstrated that *C*. *difficile* spores gain access into the intestinal epithelial cells through pathways dependent on fibronectin-α_5_β_1_ and vitronectin-α_v_β_1_ [21]. Although fibronectin, vitronectin, and integrins α_5,_ α_v,_ and β_1_ are mainly located in the basolateral membrane [40,41], we have shown that fibronectin and vitronectin are luminally accessible into the colonic mucosa of healthy young mice [21]. To date, how these molecules are increased and/or become luminally accessible in aged intestines due to increased intestinal permeability and whether this contributes to spore adherence and entry into the intestinal mucosa remains a matter of study in our research group.

We have recently shown that nystatin reduces *C*. *difficile* spore entry *in vitro* and in the ileum but not into the colonic mucosa [21]. Nystatin is a cholesterol-chelating agent that disrupts the cholesterol lipid raft required for caveolin- and integrin-dependent entry of bacterial pathogens into host intestinal cells [42,43], suggesting that caveolin may be involved in *C*. *difficile* spore internalization. This is in concordance with results reporting that senescent cells had increased levels of caveolin-1, associated with higher rates of bacterial infection. For example, *Salmonella typhimurium* entry into senescent host cells that over-express caveolin-1 exhibit increased invasion compared with non-senescent cells, and the Salmonella-entry depends on the levels of caveolin-1 expression [44]. These increased levels of caveolin in the intestinal mucosa of aged mice, coupled with a reduced mucus thickness, might also be implicated in the increased *C*. *difficile* spore-entry observed in the intestinal mucosa of older mice. Further studies attempting to address these changes will provide a comprehensive understanding of how intestinal microbes interact with the mucosa during aging and their implications in disease.

## Supporting information

S1 Fig Schematic identification of linea alba. (A) The abdominal skin of the anesthetized mouse was disinfected with 70% ethanol, then was shaved, and the skin was cleaned with povidone-iodine. (B) The incision in the skin was performed parallel to the linea alba. (C) Identification of the linea alba as a semitransparent white line in the peritoneum.
(TIF)

S2 Fig Identification of regions of interest to perform ligations between blood vessels. (A) Identification of ileum and colon using as reference the cecum. The areas of interest to be ligated are indicated by dotted lines. The yellow line and blue line denote the first and second ligation, respectively. Ligations are spaced ~1.5 cm. The ligatures with surgical silk sutures were performed between the intestine and the blood vessels. The identification of areas of interest are shown in (B) ileum and (C) proximal colon. As a reference, the first ligation was performed close to the cecum.
(TIF)

S3 Fig Tissue orientation under microscopy for mounting. Immunostained tissues were visualized under an upright light/epifluorescence microscopy to identify the tissue orientation to mount them with the luminal side up. Representative phase-contrast and Hoechst staining micrograph of (A) ileum and (B) proximal colon with the luminal or muscular side up. Scale bar 400 μm.
(TIF)

S1 Video Mouse preparation for surgery. This video shows how to anesthetize the mouse, apply ophthalmic solution, disinfect, and shave the abdomen.
(MP4)

S2 Video Midline laparotomy. This video shows how to open the abdomen skin, identify the linea alba and open the peritoneal cavity.
(MP4)

S3 Video Procedure to ligate loops. This video shows how to identify the ileum and the proximal colon, remove fecal material from the section to be ligated, identify the sites to be ligated. Also, shown how to perform the ligations without interruption of the blood vessels and injection of *C*. *difficile* spores on the ileum and colon.
(MP4)

S4 Video Midline laparotomy closure with suture. This video shows how to suture the abdominal wall and the abdominal skin with silk suture using a continuous suture technique to close the incision and let mice recover from the procedure.
(MP4)

S5 Video Extraction of the ligated loop. This video shows how to extract the ligated loop in a euthanized mouse.
(MP4)

S6 Video Washing and fixing of extracted tissues. This video shows how to open and wash the infected ligated loops and the procedure of fixing with 30% sucrose in PBS–4% paraformaldehyde.
(MP4)

S7 Video Mounting of immunostained tissues for confocal microscopy. This video shows how to orientate the tissues to put the luminal side up of the ileum and the colon, the mounting using mounting medium, and sealing it with Scotch transparent tape.
(MP4)
