# Supplementary figures and images for: Using a ligate intestinal loop mouse model to investigate Clostridioides difficile adherence to the intestinal mucosa in aged mice
Source: PLoS One. 2021 Dec 22;16(12):e0261081. doi: 10.1371/journal.pone.0261081 (PMC8694449; doi:10.1371/journal.pone.0261081)

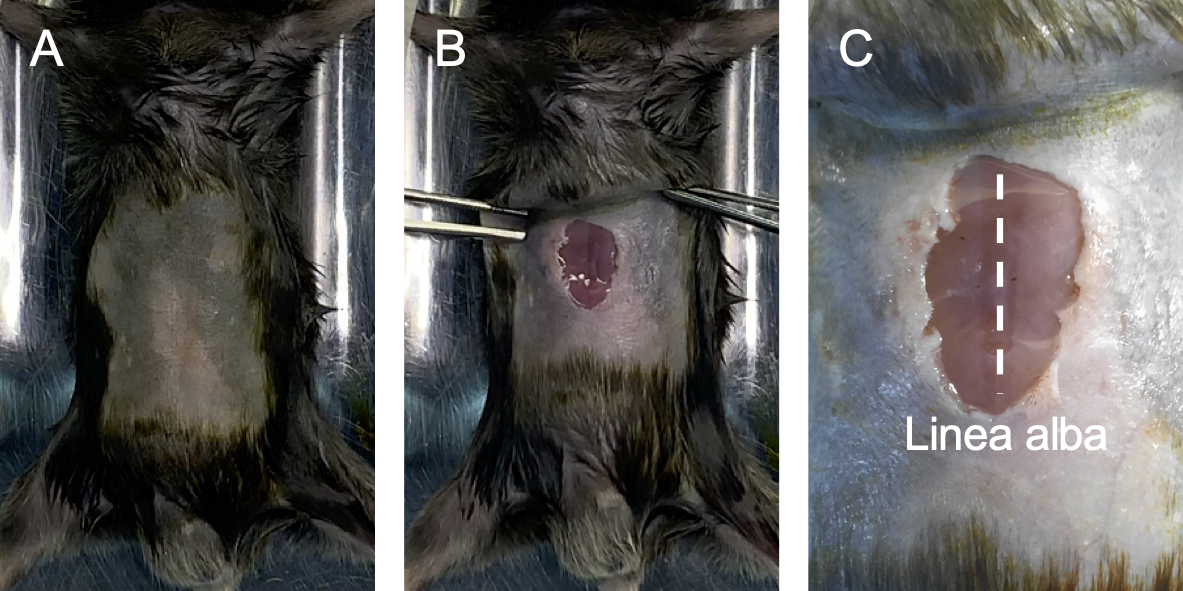

Supplement: S1 Fig — (A) The abdominal skin of the anesthetized mouse was disinfected with 70% ethanol, then was shaved, and the skin was cleaned with povidone-iodine. (B) The incision in the skin was performed parallel to the linea alba. (C) Identification of the linea alba as a semitransparent white line in the peritoneum. (TIF) [file pone.0261081.s001.tif]

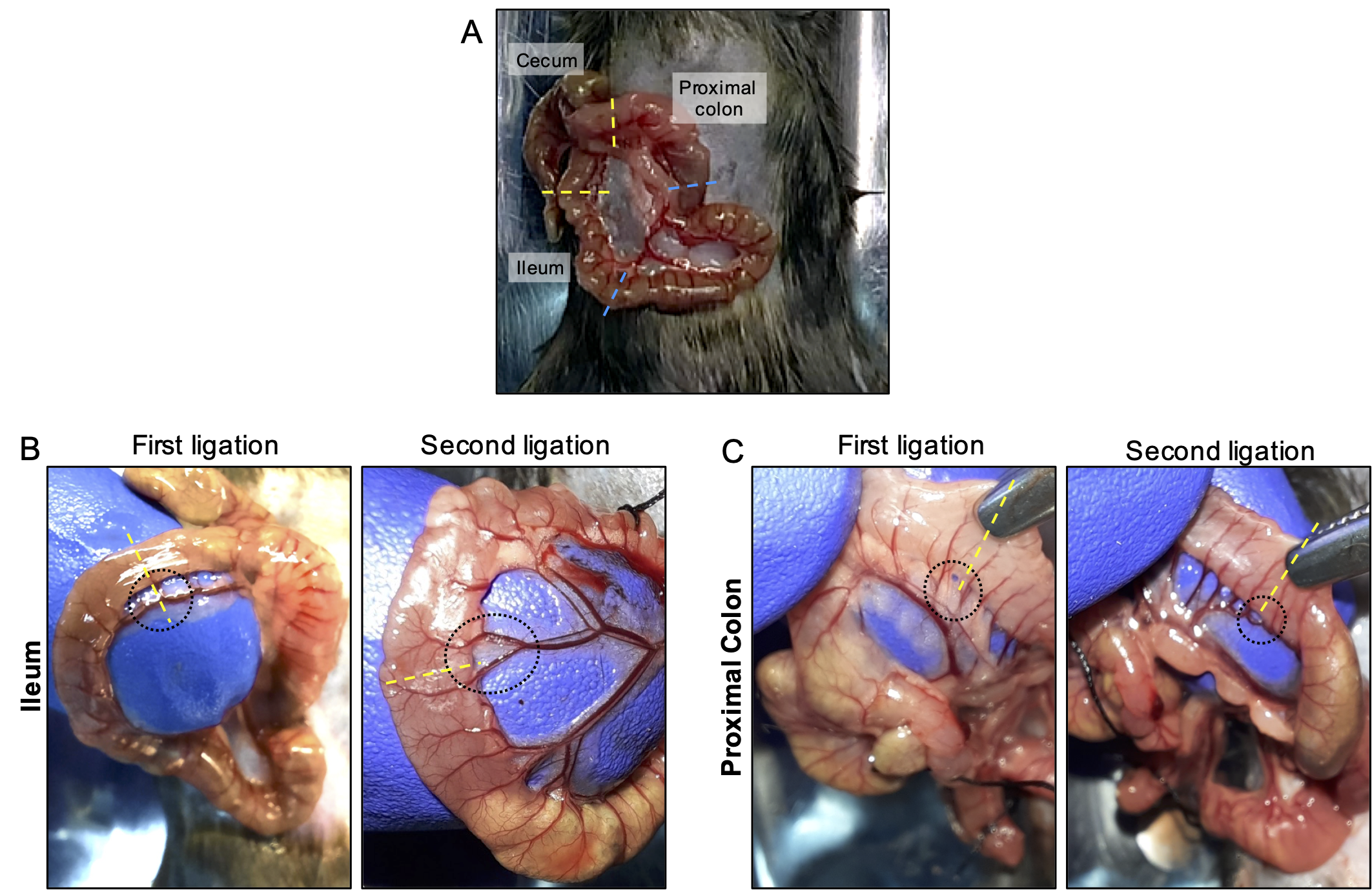

Supplement: S2 Fig — (A) Identification of ileum and colon using as reference the cecum. The areas of interest to be ligated are indicated by dotted lines. The yellow line and blue line denote the first and second ligation, respectively. Ligations are spaced ~1.5 cm. The ligatures with surgical silk sutures were performed between the intestine and the blood vessels. The identification of areas of interest are shown in (B) ileum and (C) proximal colon. As a reference, the first ligation was performed close to the cecum. (TIF) [file pone.0261081.s002.tif]

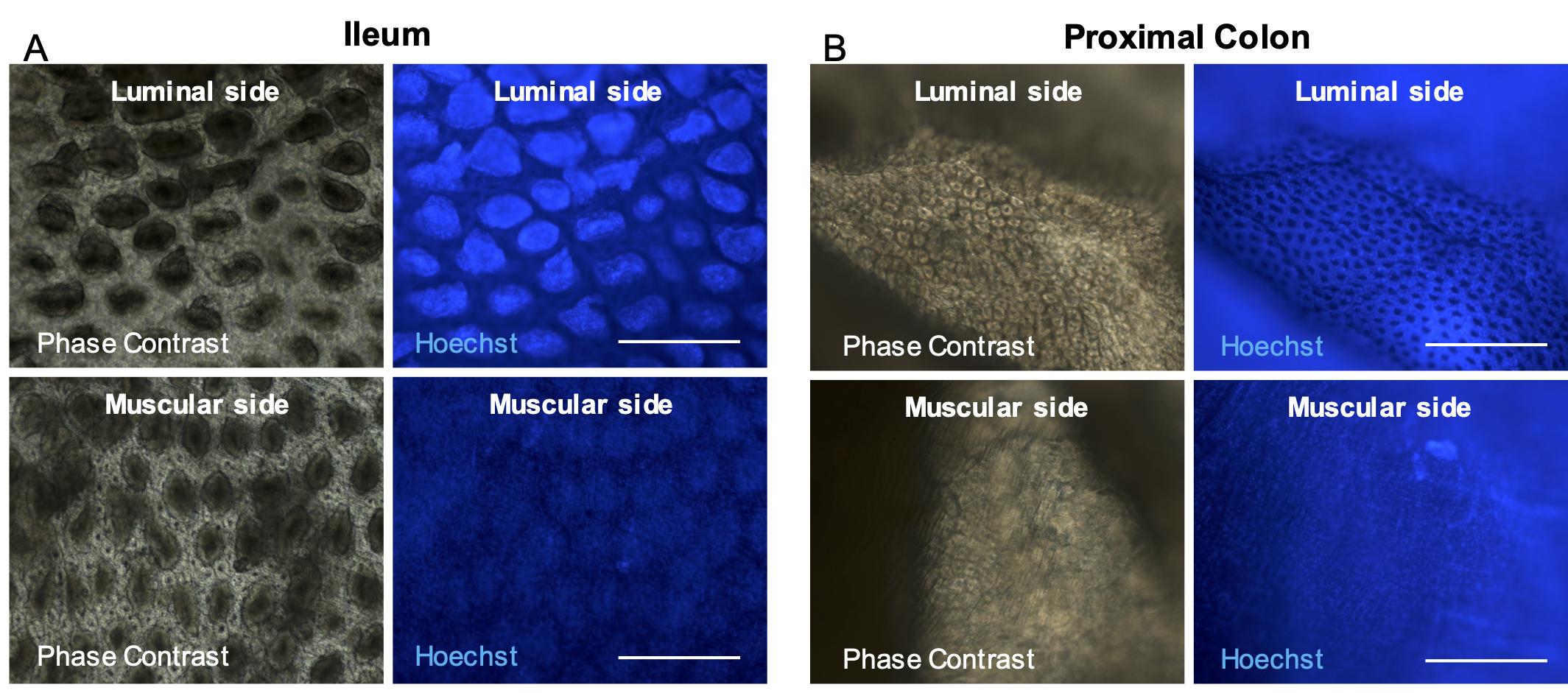

Supplement: S3 Fig — Immunostained tissues were visualized under an upright light/epifluorescence microscopy to identify the tissue orientation to mount them with the luminal side up. Representative phase-contrast and Hoechst staining micrograph of (A) ileum and (B) proximal colon with the luminal or muscular side up. Scale bar 400 μm. (TIF) [file pone.0261081.s003.tif]
